# Ten-Year Review of Gestational Trophoblastic Disease at Lady Reading Hospital, Peshawar

**DOI:** 10.7759/cureus.26620

**Published:** 2022-07-06

**Authors:** Shahzadi S Hussain, Mehnaz Raees, Rehana Rahim

**Affiliations:** 1 Department of Obstetrics and Gynecology, Lady Reading Hospital Medical Teaching Institute, Peshawar, PAK

**Keywords:** hydatiform mole, choriocarcinoma, complete mole, gestational trophoblastic neoplasia, s: gestational trophoblastic disease

## Abstract

Objective: To determine the frequency and management outcome of gestational trophoblastic disease (GTD) in Lady Reading Hospital.

Methods: This was a retrospective observational study at Lady Reading Hospital, Peshawar, from January 2011 to December 2021. Hospital records of all patients with GTD were reviewed and all were included in this study except those with an incomplete record or unconfirmed histology. Treatment was analysed in terms of surgical, chemotherapy or no treatment, and outcomes were noted in terms of complete remission, disease persistence or death.

Results: In 10 years 353 patients were admitted with GTD, and the frequency of the disease was 3.72 cases per 1000 pregnancies. The most frequent lesions were complete mole 65.2% (n=230) followed by invasive mole 20.4% (n=72). Mortality rate was 0.56% (n=2).

Maternal blood group analysis revealed that B positive 28.3% (n=100) was more frequent. O positive blood group was found more in the malignant form of the disease at 3.96% (n=14). GTD was most prevalent in 21 to 30 years of age (41.4%, n=146). Regarding treatment, in 69.97% (n=247) of cases, suction and evacuation were performed, in 4.2% (n=15) of cases hysterectomy was performed as primary therapy, and 4.8% (17) needed hysterectomy for chemoresistance.

In this study 42.49% (n=150) were given single-agent chemotherapy and 4.8% (n=17) were given multi-agent therapy. We had 21.33% (32) patients with a risk score of 7-9. In the group with a risk score of 7-9, 15.62% (n=5) patients were directly started on multi-agent therapy because of evidence of metastasis or choriocarcinoma; the remaining 84.37% (n=27) of patients who had no evidence of metastasis, no prior chemotherapy, no choriocarcinoma and International Federation of Obstetrics and Gynecology (FIGO) stage 1 were given single-agent methotrexate with folinic acid (eight days) after informed consent. In 18.75% of patients (n=6) hysterectomy was performed as the primary treatment either for haemorrhage or with age > 40, family completed, or reluctance to undergo chemotherapy. They all had a complete cure. In 3.1% (n=1) of cases, resistance to single-agent therapy was found and multi-agent treatment was started. Overall, in 96.29% of patients, complete remission was achieved with single-agent therapy in patients with risk scores of 7-9.

Conclusion: The frequency of GTD was 3.4/1000 pregnancies. Complete mole was the most frequent lesion, and single-agent chemotherapy had a good outcome in low- and high-risk patients with a risk score of 7-9 (with no evidence of metastasis, prior chemotherapy, or choriocarcinoma and FIGO stage 1).

## Introduction

Gestational trophoblastic disease (GTD) is a group of placental-related disorders derived from a pregnancy. GTD includes a spectrum of disorders, for example hydatidiform mole (complete and partial moles), invasive mole, choriocarcinoma, placental site trophoblastic tumour (PSTT) and epithelioid trophoblast tumor (ETT). Gestational trophoblastic neoplasia (GTN) is a term used to describe GTD requiring chemotherapy or excisional treatment because of the persistence of human chorionic gonadotrophin (hCG) hormone, invasion of trophoblastic tissue or presence of metastases [1].

The incidence of GTD varies worldwide. In the UK it is 1 in 714 live births, and in North America 2.5 per 1000 live births, however, the incidence is higher in Asian women as compared to non-Asian women (1 in 387 versus 1 in 752 live births) [2]. The exact incidence in Pakistan is not known because of the lack of a national registry, but different studies reported it between 0.68/1000 to 28/1000 live births [3]. In addition to ethnicity, previous history of GTN adds to the risk of having the disorder again. After one molar pregnancy, the risk of having GTD again is 1-2% and 15-20% after two previous molar pregnancies [4]. Other risk factors include extreme reproductive age, and a weak association is found with blood groups A & AB and the use of oral contraceptives for a longer duration [5].

GTD develops because of faulty fertilization which leads to abnormal proliferation of placental villi. In some cases, an empty egg is fertilized by duplicated sperm; in others, two sperms fertilise a single egg, leading to complete and partial mole. Because of the persistence of this abnormal tissue, high beta-hCG GTN can develop in different forms. Invasive moles are made up of trophoblast cells that grow into the muscle layer of the uterus. Choriocarcinoma is a malignant form of GTD and is a pure epithelial malignancy, comprising neoplastic intermediate trophoblast, cytotrophoblast, and syncytiotrophoblast elements without chorionic villi, it can spread to distant organs, most commonly lungs, brain and gastrointestinal tract. PSTT is a rare type of GTN that forms where the placenta attaches to the uterus, grows very slowly, and may take months before symptoms appear. Histologically, it is characterized by the absence of villi, and proliferation of intermediate trophoblast cells without syncytiotrophoblast cells. As syncytiotrophoblasts are absent, a low level of hCG is found in these patients. ETT is extremely rare, derived from intermediate trophoblastic cells. They often arise in the cervix or lower uterine segment, invading deeply into surrounding tissues [1].

The treatment of molar pregnancy is suction evacuation, followed by serial hCG monitoring. In cases where the hCG level remains elevated after treatment, further treatment is given in form of chemotherapy or surgery, depending upon the condition. Before starting chemotherapy, the patient is assessed using the International Federation of Obstetrics and Gynecology (FIGO) 2000 scoring system and scored as low risk (score < 6) or high risk (score >/= 7 and more) [6]. Single-agent therapy with methotrexate (MTX) and folinic acid (FA) is given for low risk while multiagent chemotherapy is prescribed for the high-risk group. The cure rate for women with a score of 6 or less is almost 100%, while the rate for women with a score of 7 or greater is 94% [7-8].

Lady Reading Hospital (LRH) is the largest hospital in Khyber Pakhtunkhwa province (KPK) and we receive most of the patients with GTD across the KPK province. This 10-year study was conducted to find the frequency of the disease in patients admitted to LRH and common risk factors among them, along with treatment outcomes.

Furthermore, the result of this study will enlighten us about disease burden and type. It will create awareness among health care providers and pregnant women on the importance of the subject leading to improvement, diagnosis, and management of these cases along with future planning.

## Materials and methods

This was a 10-year retrospective observational study at Lady Reading Hospital Peshawar. Approval from the Medical Teaching Institute Lady Reading Hospital Ethical Review Board (ref no: 322/MTI/LRH) was taken. A probability convenient sampling technique was used. We have had a registration system in place for GTD patients for more than a decade. Data were collected from hospital records of all patients admitted with the diagnosis of gestational trophoblastic disease from January 2011 to December 2021. Those cases were excluded where data is incomplete, lost in follow up or later on diagnosis was changed because of histological findings. Patient identity was kept confidential, and demographic features like age, parity, and blood group were recorded along with details of previous pregnancies, beta-hCG levels before treatment, histopathological type of GTD, size of the tumour, metastasis, staging according to FIGO (stage 1: GTD are completely restricted to the corpus of the uterus, stage II: GTD involving vagina or adnexa but is restricted to the genital structures, stage III: GTDs developing to the lungs may involve genital tract, stage IV: All other metastatic sites) was noted along with risk scoring according to FIGO score 2000 with slight modification (discussed later); treatment was given and analysed in terms of medical or surgical. A single course of medical treatment consisted of methotrexate (MTX) based on the eight-day protocol consisting of 1 mg/kg MTX in combination with 0.1 mg/kg FA every other day followed by six days rest period. Stable or increasing beta-hCG levels after two courses of treatment were considered a failure to initial chemotherapy. Patients were switched to a multi-agent therapy: EMA-CO (etoposide, methotrexate, actinomycin D, cyclophosphamide, oncovin). Surgical treatment was noted in terms of suction and evacuation or hysterectomy. The outcomes were noted in terms of complete recovery, failed first-line treatment and the death of the patient.

Patients were divided according to high risk or low risk. The high-risk group was further divided into two groups, those with a risk score of ≥7 with evidence of choriocarcinoma, metastasis, prior single-agent chemotherapy and stage II or higher FIGO staging and those with a risk score of more than 9 with or without previously mentioned risk factors. All these patients received multi-agent chemotherapy from start. The low-risk group had a risk score of ≤6 received single-agent therapy, and patients with a risk score of 7-9 (with no choriocarcinoma, no metastasis, previous single-agent chemotherapy and stage I FIGO staging) after informed written consent were also treated with single-agent therapy with fortnightly beta-hCG monitoring. This slight modification was in practice for a long time in our hospital, based on good results from previous research by Fatima et al. and Elit et al. [9,10].

Patients were followed with beta-hCG levels fortnightly, complete remission was confirmed once three consecutive samples were normal with monthly follow-up for the next six months was done to ensure complete recovery and rule out relapse.

Data were entered and analysed using SPSS version 2.0 (IBM Corp., Armonk, NY, USA). Frequencies and percentages were used to describe categorical variables, results were presented in tables and graphs, a post-stratification p-value was applied and a p-value of <0.05 was considered significant.

## Results

In this 10-year study a total 353 patients were admitted with GTD; the prevalence of disease was 3.72 cases per 1000 pregnancies. Frequency of complete mole was 65.2% (n=230), partial mole 10.5% (n=37), invasive mole 20.4% (n=72), choriocarcinoma 3.1% (n=11) and placental site trophoblastic tumor was 0.6% (n=2). Mortality rate was 0.56% (n=2).

**Figure 1:**
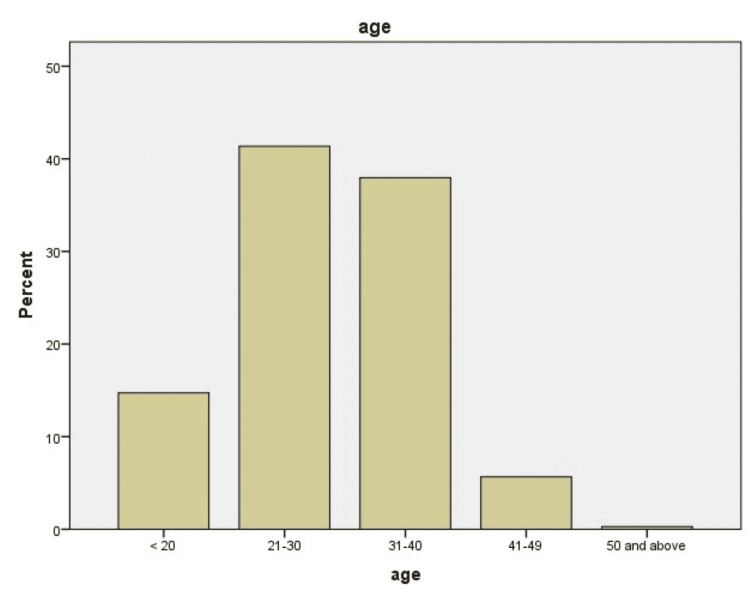
Percentage of gestational trophoblastic disease (GTD) patients according to age

**Figure 2:**
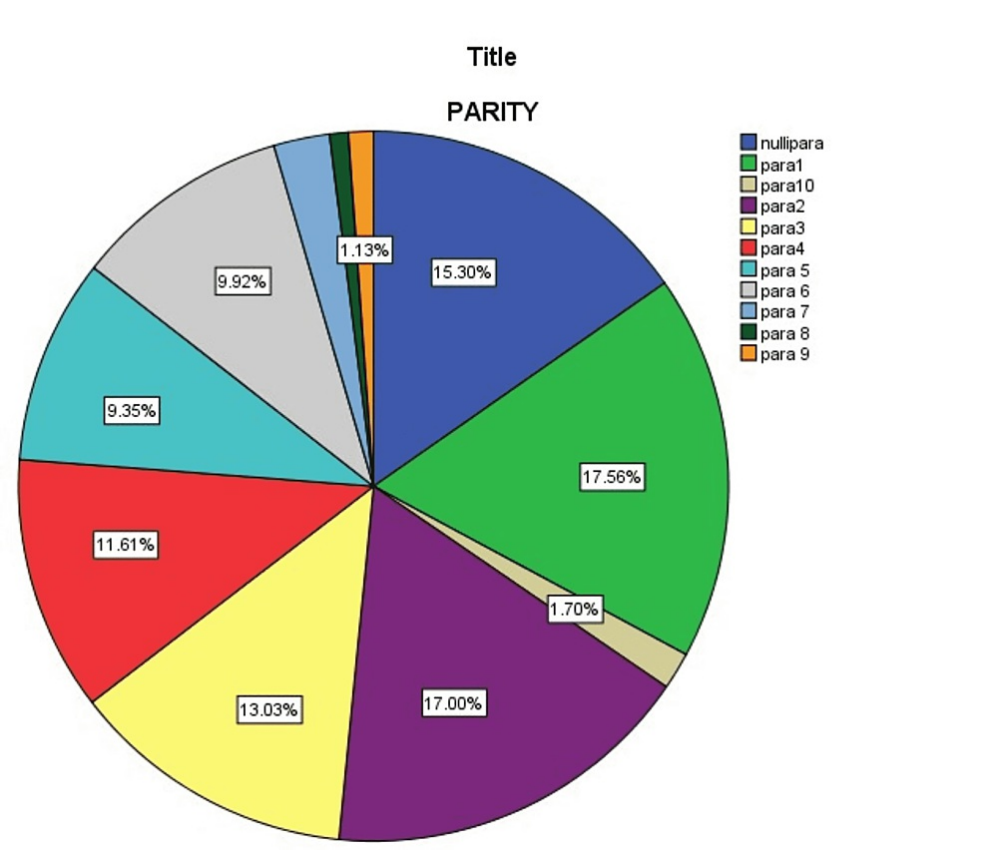
Parity of patients with gestational trophoblastic disease (GTD) (in percentages)

GTD was most prevalent in the 21-30 age group (41.4%, n=146) (Figure 1), however the malignant form of the disease was more common in 31-40 years of age (Table 1). Maternal blood group analysis revealed that 80.16% (n=336) with GTD had RH positive antigen. B positive blood group was more frequent in 28.3% (n=100) of patients. However O positive blood group was found more in the malignant form of the disease (invasive mole 3.96% (n=14), and choriocarcinomas 4) (Table 2). GTD was found more prevalent in patients with low parity (Figure 2). Overall beta-hCG level of more than 10,000 - 100,000 was found to be more prevalent (34.3%) (Table 3). In this study, suction and evacuation was performed in 69.97% of patients, hysterectomy was performed in 4.2% (n=15) as primary treatment, and hysterectomy was needed in 4.8% (n=17) of patients for chemoresistance (Table 4).

**Table 1:** Age of patient and type of lesion PSTT: placental site trophoblastic tumour

			Type of lesion					Total
			complete mole	incomplete mole	invasive mole	choriocarcinoma	PSTT	
age	< 20	Count	43	2	6	1	0	52
		% within type of lesion	18.7%	5.4%	8.3%	9.1%	0.0%	14.7%
	21-30	Count	96	20	25	3	2	146
		% within type of lesion	41.7%	54.1%	34.7%	27.3%	66.7%	41.4%
	31-40	Count	83	14	31	5	1	134
		% within type of lesion	36.1%	37.8%	43.1%	45.5%	33.3%	38.0%
	41-49	Count	8	1	10	1	0	20
		% within type of lesion	3.5%	2.7%	13.9%	9.1%	0.0%	5.7%
	50 and above	Count	0	0	0	1	0	1
		% within type of lesion	0.0%	0.0%	0.0%	9.1%	0.0%	0.3%
Total		Count	230	37	72	11	3	353
		% within type of lesion	100.0%	100.0%	100.0%	100.0%	100.0%	100.0%

**Table 2:** Blood group and type of lesion crosstabulation PSTT: placental site trophoblastic tumour

Blood group			Type of lesion					Total
			complete mole	incomplete mole	invasive mole	Choriocarcinoma	PSTT	
	missing	Count	12	1	4	0	0	17
		% within type of lesion	5.2%	2.7%	5.6%	0.0%	0.0%	4.8%
	A+	Count	41	4	10	3	0	58
		% within type of lesion	17.8%	10.8%	13.9%	27.3%	0.0%	16.4%
	A-	Count	20	3	4	2	0	29
		% within type of lesion	8.7%	8.1%	5.6%	18.2%	0.0%	8.2%
	B+	Count	57	9	31	2	1	100
		% within type of lesion	24.8%	24.3%	43.1%	18.2%	33.3%	28.3%
	B-	Count	12	4	2	0	0	18
		% within type of lesion	5.2%	10.8%	2.8%	0.0%	0.0%	5.1%
	AB+	Count	24	3	6	0	0	33
		% within type of lesion	10.4%	8.1%	8.3%	0.0%	0.0%	9.3%
	AB-	Count	3	0	0	0	0	3
		% within type of lesion	1.3%	0.0%	0.0%	0.0%	0.0%	0.8%
	O+	Count	60	12	14	4	2	92
		% within type of lesion	26.1%	32.4%	19.4%	36.4%	66.7%	26.1%
	O-	Count	1	1	1	0	0	3
		% within type of lesion	0.4%	2.7%	1.4%	0.0%	0.0%	0.8%
Total		Count	230	37	72	11	3	353
		% within type of lesion	100.0%	100.0%	100.0%	100.0%	100.0%	100.0%

**Table 3:** Risk factors for gestational trophoblastic disease (GTD) b-hCG: beta-human chorionic gonadotropin

		Frequency	Percentage
b-hCG level before treatment	< 1000	55	15.5
	1000- 10000	75	21.2
	10000- 100000	121	34.3
	>100000	102	28.9
	Total	353	100.0
Preceding pregnancy	Nullipara	40	11.3
	Molar	11	3.1
	Miscarriage	119	33.7
	Term	183	21.8

**Table 4:** Type of treatment received by patients EMA-CO: etoposide, methotrexate, actinomycin D, cyclophosphamide, oncovin; S&E: suction and evacuation

Type of treatment		Number of patients	Percentage
Surgical treatment	No surgical intervention	74	20.96
	S&E	247	69.97
	Hysterectomy	15	4.2
	Hysterectomy after failed chemotherapy	17	4.8
	Total	353	100.0
Chemotherapy	No chemotherapy given	180	51
	Single-agent	150	42.49
	EMA-CO	17	4.8
	Single-agent failed Referred for multiagent	6	1.7
Total		353	100.0

In this study 42.49% (n=150) were given single-agent therapy, in 96% (n=144) of cases single-agent therapy was successful, however in 4% (n=6) single-agent failed and patients were referred for multi-agent therapy, among them one patient died during treatment, the rest recovered. Multi-agent therapy was given in 4.8% (n=17) of patients after initial diagnosis because of a risk score of more than 9.

Single-agent therapy was given for a score of 6 or less, while multi-agent was given to all patients with a score of 1 or above. We had 21.33% (32) patients with risk scores between 7-9. In the group with a risk score of 7-9, 15.62% (n=5) patients were directly started on multi-agent therapy because of evidence of metastasis or choriocarcinoma; the remaining 84.37% (n=27) patients who had no evidence of metastasis, no prior chemotherapy, no choriocarcinoma and FIGO stage 1 were given single-agent chemotherapy after informed written consent. In 18.75% (n=6) of patients, hysterectomy was performed as a primary treatment either for haemorrhage or other reasons (age > 40, family completed and reluctance to undergo chemotherapy). They all had a complete cure. In 3.1% (n=1) of cases there was resistance to single-agent therapy and multi-agent treatment was started. Overall, complete remission was achieved in 96.29% of patients with single-agent therapy in patients with a risk score of 7-9 (Table 5).

**Table 5:** Risk score and chemotherapy crosstabulation (p-value <0.001) EMA-CO: etoposide, methotrexate, actinomycin D, cyclophosphamide, oncovin

Risk score		Chemotherapy				Total
		single agent	Multiagent EMA-CO	no chemotherapy given	single agent failed, referred for multiagent	
	1	3	0	0	0	3
	2	21	2	22	0	45
	3	29	3	49	0	81
	4	29	2	34	1	66
	5	23	3	38	0	64
	6	26	1	31	3	61
	7	9	2	4	0	15
	8	9	3	2	0	14
	9	1	0	1	1	3
	10	0	1	0	0	1
	Total		150	17	181	6	353

In this study, pre-treatment beta-hCG level ≥1000 was observed more frequently with invasive mole while beta-hCG of 10,000 and above was found to be more common in patients with choriocarcinoma and patients with failed chemotherapy (Table 6).

**Table 6:** b-hCG level before treatment and type of lesion crosstabulation (p-value <0.001) b-hCG: beta-human chorionic gonadotropin; PSTT: placental site trophoblastic tumour

			Type of lesion					Total
			complete mole	incomplete mole	invasive mole	Choriocarcinoma	PSTT	
b-hCG level before treatment	< 1000	Count	22	4	27	2	0	55
		% within type of lesion	9.6%	10.8%	37.5%	18.2%	0.0%	15.6%
	1000- 10000	Count	43	6	24	0	2	75
		% within type of lesion	18.7%	16.2%	33.3%	0.0%	66.7%	21.2%
	10000- 100000	Count	85	17	14	4	1	121
		% within type of lesion	37.0%	45.9%	19.4%	36.4%	33.3%	34.3%
	>100000	Count	80	10	7	5	0	102
		% within type of lesion	34.8%	27.0%	9.7%	45.5%	0.0%	28.9%
Total		Count	230	37	72	11	3	353
		% within type of lesion	100.0%	100.0%	100.0%	100.0%	100.0%	100.0%

Two patients died during the course of treatment. One of them had a complete mole; S&E was performed, risk score <6, rising beta-hCG trend in follow-up, started on single-agent chemotherapy. As her beta-hCG level continued to rise, the patient was shifted to multi-agent therapy. She received only one cycle of EMACO; beta-hCG dropped but she was lost in follow-up. Four months later she presented with heavy vaginal bleeding choriocarcinoma with extensive metastasis to the chest and liver. A hysterectomy was performed but she died within a week. Another patient presented with choriocarcinoma with metastasis to the lungs, liver, and brain, and was started on multi-agent therapy (EMACO) but died during chemotherapy. 

## Discussion

The gestational trophoblastic disease is unique in the way that it affects patients of reproductive age and has a very good prognosis if diagnosed and managed in time. It has a variable prevalence in different parts of the world, with Pakistan among the areas of high prevalence. In our study, it affected ladies with low parity, between 21-30 years of age, with RH positive and blood group B being most common among patients with GTD. Complete moles were followed by invasive moles in terms of frequency. Single-agent therapy with MTX and folinic acid treatment was given to selected patients with risk scores up to 9, with only 1.7% requiring multi-agent and 29.4% requiring hysterectomy for failed chemotherapy with single and multi-agent therapy.

The prevalence of GTD reported in this study (3.72 cases per 1000 pregnancies) correlated with findings from a related study in Karachi (3.891/1000) [11], nevertheless, this is lower than a related 10-year study conducted in Nigeria (5.7/per 1000 pregnancies) [12] but far higher than a 20-year study in the Netherlands (1.67 per 1000 deliveries) [13]. The high prevalence may be due to an increase in genetic predisposition or poor nutritional status of people in this part of the world; this aspect is not explored till recently in Pakistan. 

Regarding the type of lesion in this study, complete moles were most frequent (65.2%). A similar study in Nigeria reported 57.34% complete mole [12], and in another seven-year study in Oman complete hydatidiform mole was reported in 43.8% [14]. However, in our study the second most frequent lesion was invasive mole (20.4%), which is higher than reported in similar regional and international studies (1.46%, 2.7%, 2.56% and 12.5%) [15-18]. This is a very unusual finding and shows a high rate of progression of the benign lesion into malignant form in this part of the world. One of the reasons for this could be the delayed presentation of patients from far-flung areas, who had initial substandard treatment by inexperienced birth attendants.

Age analysis in this study revealed that overall, most patients were between 21-30 years of age (41.4%). Related studies in Pakistan and India showed the same age group to have a high prevalence (54.54% and 66%), however international studies reported a high prevalence in 31-40 years of age [14-18]. This is an important finding which shows that a much younger population is affected in this part of the world. One of the reasons can be the high incidence of early marriages. Regarding the malignant form of the disease in our study, it was more prevalent in 31-40 years of age (invasive mole 43.1 % and choriocarcinoma 45.6%). The reason can be an effect of aging on eggs and increased chances of abnormal fertilization in advanced age.

In this study 80.16% of patients with GTD had RH positive antigen. Jagtap et al. [15] reported similar findings (83.11%) and overall blood groups B (33.4%) and O (26.9%) were the most common blood groups in our study, while in a study in Karachi [11] blood group A was reported to be more prevalent (OR=1.6, CI=I.01-2.53); however, international studies found blood group O to be more prevalent (97.8% and 79.3%) [19-20]. In this study blood group, O was found more commonly in the malignant form of disease only. This aspect is not well explored in previous studies on the same topic.

We observed that patients with low parity (i.e., nullipara 15.3%, para-one 17.6% and para two 17.0%) were the major group with GTD. Similar results were found in a regional study at Abbottabad (42% para 0-1) [16], a study in Tanzania 65% of patients were para 0-1 and studies by Fatima et al., Parazzini F et al., Brinton et al., and Saraf and Ghodke, which shows that risk of GTD decreases with increase in parity [9,20-23].

Pre-treatment beta-hCG level ≥1000 was observed more frequently with invasive mole. Mousavi et al. [24] in their study elaborated similar findings that pre-evacuation beta-hCG level may be lower in GTN as compared to benign form, however, beta-hCG of 10,000 and above was found to be more common in patients with choriocarcinoma and patients with failed chemotherapy.

In this study suction and evacuation (S&E) was performed in 69.97% of cases. A similar study in Quetta showed the same results (72.9%) [9], while the one in Sind reported slightly higher figures (82.6%) [25]. This shows that S&E is the main modality of treatment of GTD. Hysterectomy was performed in 4.2% of patients as initial treatment; the majority of them were more than 40 years with family completed, had a risk score of more than 7, and were not willing for chemotherapy and prolonged follow-ups. Makhathini et al. [26] in their study reported hysterectomy in 4.8% of patients as a primary surgical treatment. Single-agent chemotherapy was given to 42.49% of patients (complete cure), and 1.7% did not respond to single-agent treatment and were referred for multi-agent therapy. Makhathini et al. reported 3.2% resistance to single-agent while hysterectomy for failed chemotherapy was performed in 4.8% of patients. These results are far less than the figure reported by Capobianco et al. (29.17%) [27]. In 51% of patients beta-hCG reverted to normal without any chemotherapy, and an Italian study showed the same figures.

In this 96.29% of patients, complete remission was achieved with single-agent therapy in patients with risk scores of 7-9. Our study is the first of its kind in reporting this aspect. Literature review shows a paucity of studies where a single agent is used precisely with a risk score of 7-9 (with the exclusion of high-risk factors). MTX is relatively safe, easily available, cost-effective and has fewer side effects as compared to multi-agent therapy and can be given in local centers. In this part of the world, it is sometimes difficult for patients to afford and reach specialized centers for multi-agent therapy and some are reluctant to have it because of concerns regarding chemotherapy, so with careful patient selection, MTX with folinic acid can be considered in patients with scores of 7-9. However, we had a small sample size of groups 7-9, so more research is recommended on the subject to draw conclusive evidence.

The limitation of this study was the retrospective nature of the study and retrieval of patient records over 10 years.

## Conclusions

GTD is a common disease in this part of the world. It affects a relatively young population of women with low parity. Complete mole followed by invasive mole was the most frequent lesion in this study. It is important to individualize treatment for patients with GTD depending upon risk factors, risk score, type of lesion, metastasis, and patient wishes. Less toxic single agent (methotrexate with folinic acid) is the preferred mode of treatment in most cases, while multi-agent therapy is reserved for high-risk patients only.
